# Tolerance of Stored Boar Spermatozoa to Autologous Seminal Plasma: A Proteomic and Lipidomic Approach

**DOI:** 10.3390/ijms21186474

**Published:** 2020-09-04

**Authors:** Lisa Höfner, Anne-Marie Luther, Alessandra Palladini, Thomas Fröhlich, Dagmar Waberski

**Affiliations:** 1 Unit for Reproductive Medicine of Clinics/Clinic for Pigs and Small Ruminants, University of Veterinary Medicine, 30559 Hannover, Germany; lisa.hoefner@tiho-hannover.de (L.H.); anne-marie.luther@tiho-hannover.de (A.-M.L.); 2 Paul Langerhans Institute Dresden, Helmholtz Zentrum München, University Hospital and Faculty of Medicine Carl Gustav Carus, Technische Universität Dresden, 01307 Dresden, Germany; alessandra.palladini@tu-dresden.de; 3 German Centre for Diabetes Research (DZD e.V.), 85764 Neuherberg, Germany; 4 Laboratory for Functional Genome Analysis (LAFUGA), Gene Center, Ludwig-Maximilians-University Munich, 81377 Munich, Germany; frohlich@genzentrum.lmu.de

**Keywords:** boar seminal plasma, sperm quality, semen preservation, biomarker, proteomics, lipidomics

## Abstract

Long-term exposure of liquid preserved boar spermatozoa to seminal plasma (SP) can cause dramatic sperm injury. This study examined whether boar specificity exists in the sensitivity of spermatozoa to SP and whether correspondent biomarkers can be identified. Consecutive ejaculates (*n* = 4–5) collected from 19 boars were centrifuged, diluted with a pH-stablising extender with 10% (*v*/*v*) autologous SP and evaluated by computer-assisted semen analysis and flow cytometry. Up until 144 h storage, four boars showed consistently high sperm motility, viability and mitochondria activity, and one boar showed consistently low values. Intra-boar variability was high in the other boars. Screening of SP (*n* = 12 samples) for protein markers using mass spectrometry identified three protein candidates of which the granulin precursor, legumain and AWN were 0.5 to 0.9 log2-fold less abundant (*p* < 0.05) in SP-resistant compared to SP-sensitive samples. Lipidome analysis by mass spectrometry revealed 568 lipids showing no difference between the SP-groups. The most abundant lipids were cholesterol (42,442 pmol), followed by phosphatidylserine (20,956 pmol) and ether-linked phosphatidylethanolamine (13,039 pmol). In conclusion, three candidate proteins were identified which might be indicative of SP-tolerance of sperm during long-term storage. Noteworthy, a first lipidomic profile of boar SP is presented.

## 1. Introduction

In the porcine species, liquid-stored semen are used in 99% of artificial inseminations (AI) because the quality of cryopreserved semen is still low. The success of AI depends on the identification and selection of boars with high fertility and suitability for semen preservation. There is an ongoing trend to increase AI efficiency towards prolonged storage periods and lower sperm numbers per semen dose [1]. Compared to other species, the boar ejaculate exhibits a large volume, varying between 200 and 500 mL. A high volume is often associated with a low sperm concentration in the native semen, which results in a relatively high amount of seminal plasma (SP) in the semen portion. During natural mating, sperm are quickly separated from the SP in the female genital tract [2]. Consequently, there is an unphysiological prolonged exposure of sperm to SP during in vitro storage for several days. Previous studies demonstrated a dose- and time-dependent depressing effect of boar SP on motility, which becomes increasingly apparent after 72 h of storage [3,4]. Thus, removal of SP prior to long-term liquid storage can be beneficial for maintaining sperm motility [3] and fertilising ability [5], but seems not to be applicable in AI practice. Ejaculates from different boars differ in their sperm’s sensitivity to autologous SP [3]. This suggests that the inter-boar difference to withstand semen storage stress [6,7] could at least partially reside in an individual tolerance of boars to their own SP. In fact, the qualitative and quantitative composition of SP does not only vary among species, but also between males within a species and between ejaculate fractions [8,9]. Furthermore, this is influenced by environmental factors [10]. Moreover, a low sperm concentration in the semen dose makes sperm more susceptible to adverse effects of SP during in vitro storage [3] and a protective extender can counteract the SP-induced loss of sperm function [4].

It can be supposed that a great part of the complexity in sperm-SP interaction is caused by differences in the composition of the SP, particularly proteins and lipids. This could originate from the absence, presence or concentration of some SP-components [11]. Proteins are the bulk components of the SP and mainly built by the spermadhesin family. These multifunctional proteins have various effects on spermatozoa and the female genital tract, like regulation of sperm capacitation, gamete interaction, immunomodulation and formation of the oviductal sperm reservoir [12,13]. Besides this value for fertility, sperm-damaging effects were shown for the AQN-3 homologue and other heparin-binding spermadhesins [14,15], and presumably other secretions from the post sperm-rich fraction. Proteomic data of boar SP are now available [16] and links to semen preservability and fertility are being established [17]. Quantitative expression of particular SP proteins differs between ejaculate fractions [17,18,19] and is associated with differences in sperm quality [20,21,22], sperm freezability [23] and fertility [16,24,25]. Studies about SP-proteins as biomarkers for long-term liquid storage of boar spermatozoa, however, are sparse. Recently, Barranco et al. [26] demonstrated a predictive value for seven SP-cytokines for the motility of liquid preserved boar sperm after 144 h of storage although the mode of action remains to be shown.

Lipids are a basic component of semen, contributing to the membrane structure, metabolism of spermatozoa and their ability to capacitate and fertilise the female gamete [27]. There are currently limited descriptions of lipids in the porcine SP. Until now, mass spectrometry-based shotgun lipidomics of SP have been reported for stallions [28] and humans [29], but data for boars are lacking.

Identifying potential biomarkers in SP for long-term semen preservation requires in-depth knowledge about inter- and intramale variability as well as recognition of detrimental SP components. The first aim of the present study was to examine whether a boar specificity exists in the sensitivity of stored sperm to autologous SP. The second aim was to expand our knowledge on the boar SP lipidome and to relate differences in semen preservability to proteomic and lipidomic profiles of SP.

## 2. Results

### 2.1. Effect of Different Concentrations of SP on Stored Spermatozoa

**Figure 1:**
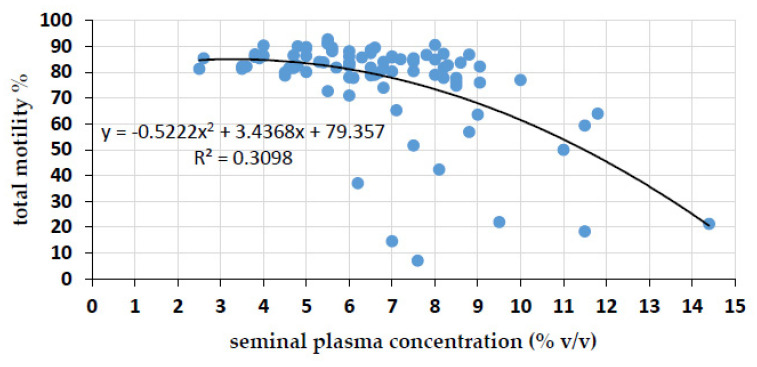
Sperm motility in relation to the concentration of seminal plasma in the semen dose. Pearson’s correlation (*r* = −0.52, *p* < 0.0001) between sperm motility at 144 h of storage and the concentration of seminal plasma in uncentrifuged semen portions extended to 18 × 10^6^ sperm/mL in 100 mL volume; *n* = 87 semen samples from 19 different boars.

**Figure 2:**
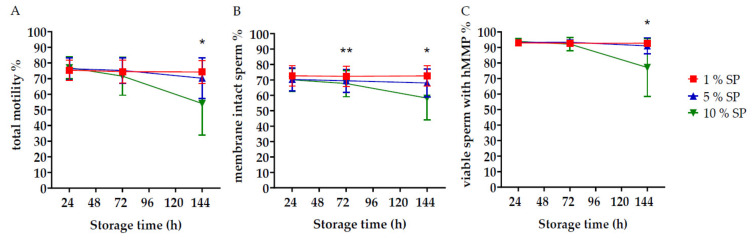
Sperm quality in stored semen doses containing different concentrations of seminal plasma (SP). Sperm pellets of centrifuged ejaculates (*n* = 4–5 from each of 19 boars) were extended with modified Beltsville thawing solution (mBTS) to 18 × 10^6^ sperm/mL containing three different concentrations of SP: 1%, 5% and 10% (*v*/*v*; final concentration) and semen portions were stored at 17 °C. Graphs present in **A**) total motility (%), **B**) membrane intact (%; propidium iodide negative and peanut agglutinin (PNA) negative) sperm (%); and **C**) viable sperm (%) with high mitochondria membrane potential (hMMP) (propidium iodide-negative sperm with J-aggregates). Values are presented as means and standard deviation (± SD). *: 10% SP differs from 5% and 1% SP (*p* < 0.05); **: 10% SP differs from 1% SP (*p* < 0.01).

Sperm total motility in uncentrifuged control semen portions at 144 h of storage amounted to 76.8 ± 14.7% and was negatively correlated (*r* = −0.522, *p* < 0.0001) to the amount of SP in the semen portions (Figure 1). Sperm data of centrifuged samples with defined amounts of SP during storage are presented in Figure 2. Storage length and SP concentration affected sperm motility, viability and mitochondria activity (*p* < 0.001; Figure 2). At 144 h, semen samples with 10% (*v*/*v*) SP exhibited a significant decrease (*p* < 0.01) in total motility (54.1 ± 20.3%), sperm viability (58.2 ± 14.1%) and viable sperm with high mitochondria membrane potential (hMMP; 77.1 ± 18.6%) when compared to 24 h and 72 h of storage. In the other two sample groups containing 1 and 5% (*v*/*v*) SP, sperm kinematics did not differ (*p* > 0.05) between the storage periods. At 72 h of storage, the percentage of sperm with intact membranes was significantly lower in the presence of 10% (*v*/*v*) SP (67.6 ± 17) compared to samples with 1% (*v*/*v*) SP (72.3 ± 17). There was no difference (*p* > 0.05) in the total motility and mitochondria activity between samples with 1% (*v*/*v*) and 10% (*v*/*v*) SP at 72 h of storage. At 144 h, sperm kinematics were significantly higher (*p* ≤ 0.01) in the presence of 1% (*v*/*v*) and 5% (*v*/*v*) SP compared to 10% (*v*/*v*) SP. After 72 h of storage and subsequent thermic incubation, the total motility in samples with the highest SP concentration was lower (40.9 ± 14.8%; *p* < 0.001) compared to semen samples with 1% (*v*/*v*) SP (68.2 ± 8.8%) and 5% (*v*/*v*) SP (58.9 ± 13.7%); semen samples with 5% (*v*/*v*) SP showed a significantly lower motility (*p* < 0.01) compared to semen samples with 1% (*v*/*v*) SP. Data are shown in Figure S1.

### 2.2. Boar Specificity and Ejaculate Effects

**Figure 3:**
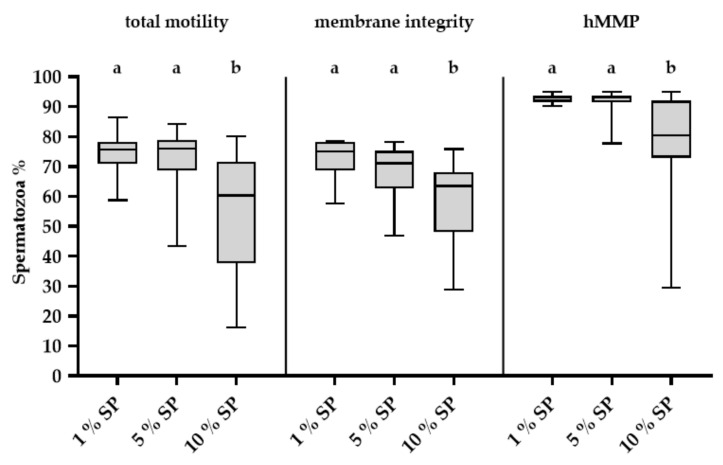
Sperm quality after 144 h of storage in semen doses containing different concentrations of seminal plasma (SP). Box-whisker plots showing variation in the total motility (%), membrane intact (% propidium iodide negative and peanut agglutinin (PNA) negative) sperm (%), and viable sperm (%) with high mitochondrial membrane potential (hMMP) (propidium iodide negative sperm with J-aggregates). Semen samples contain three different final concentrations of autologous SP: 1%, 5% and 10% (*v*/*v*). Different letters indicate significant difference (*p* < 0.01).

**Figure 4:**
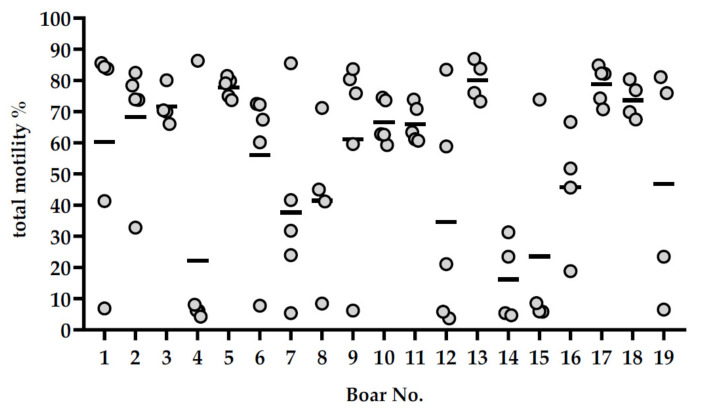
Sperm motility after 144 h of storage in single semen doses containing 10% (*v*/*v*) seminal plasma. Scatterplot showing the total motility (%) of semen samples with 10% autologous seminal plasma collected from 19 boars (*n* = 4–5 ejaculates per boar). Dots show the values measured in each sample and the bars show the mean for each boar.

At 144 h of storage, a high variation in sperm quality values was noticed in semen samples with 10% (*v*/*v*) SP (Figure 3). At 144 h with 10% (*v*/*v*) SP, samples of four resistant boars, Nos. 5, 13, 17 and 18, showed consistently high sperm motility (≥ 65%) (Figure 4), viability (≥ 65%) and mitochondria activity (≥ 90%) (Figure S2), whereas one boar (No. 14) showed consistently low values for these traits. The variability in sperm total motility, membrane integrity and mitochondria activity values among boars (inter-boar variability) and between ejaculates within boars (intra-boar variability) were evaluated in 87 semen samples from the 19 boars (*n* = 4–5 ejaculates per boar). The inter-boar variability was lower than the intra-boar variability, as shown by intra class coefficients (ICCs) of 0.7 for motility, 0.65 for membrane integrity and 0.68 for hMMP. Nevertheless, the ejaculate had a significant effect (*p* < 0.01) on total motility after thermic stress and membrane integrity of the stored samples, but no effect (*p* > 0.05) on total motility and mitochondria membrane potential after storage.

### 2.3. Proteomics of SP

**Figure 5:**
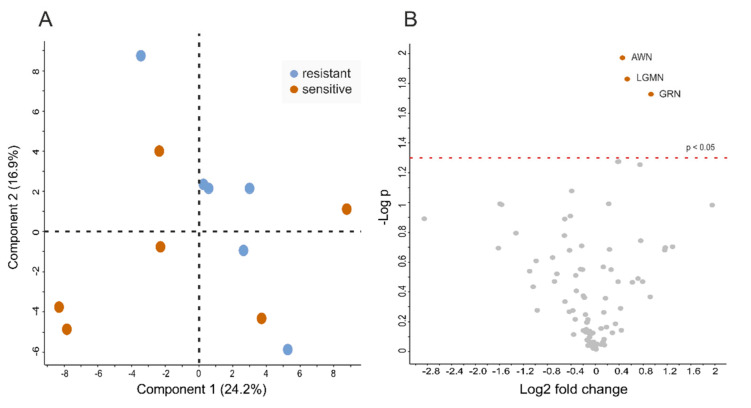
Exploratory data analysis of proteins identified in seminal plasma of boars showing high sperm quality (resistant) and low sperm quality (sensitive) in stored semen doses containing 10% (*v*/*v*) autologous seminal plasma. Overview of the results from the LC-MS/MS analysis of boar seminal plasma proteins. (**A**) Principal component analysis (PCA) of normalised protein intensity values shows no distinct separation of the two groups, whereas (**B**) volcano plot analysis reveals three differently abundant protein candidates (*p* < 0.05) in seminal plasma samples collected from ejaculates with resistant (blue boxes) and sensitive (orange boxes) sperm.

**Figure 6:**
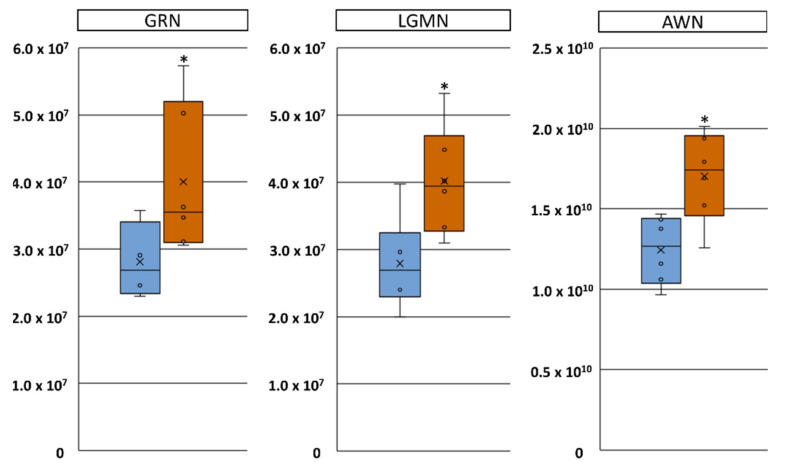
Proteins being significantly altered in abundance in seminal plasma of boars showing high sperm quality (resistant) and low sperm quality (sensitive) in stored semen doses containing 10% (*v*/*v*) autologous seminal plasma. Box plots of granulin (GRN), legumain (LGMN) and AWN intensity values in seminal plasma samples collected from ejaculates with resistant (blue boxes) and sensitive (orange boxes) sperm. * *p* < 0.05.

LC-MS/MS analysis of SP (*n* = 6 from resistant samples and *n* = 6 from sensitive samples) led to the quantification of 230 proteins at a false discovery rate < 1%. The principal component analysis (PCA) showed no distinct separation between ejaculates of long-term stored resistant and sensitive samples (Figure 5A). However, the volcano plot analysis (Figure 5B) and Students t-tests (Figure 6) revealed three proteins to be significantly altered in abundance. In particular, granulin precursor (GRN) (log2-fold change = 0.9; *p*-value = 0.02), legumain (LGMN; log2-fold change = 0.54; *p*-value = 0.01) and AWN (log2-fold change = 0.45; *p*-value = 0.01) were less abundant in SP-resistant compared to SP-sensitive samples.

### 2.4. Lipidomics of SP

**Figure 7:**
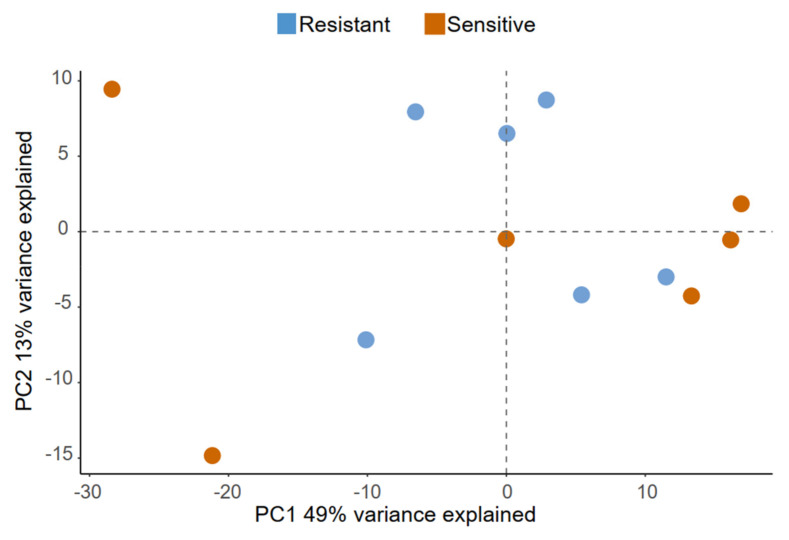
Exploratory data analysis of lipids identified in seminal plasma. Principal component analysis (PCA) showing how seminal plasma samples map onto principal components 1 and 2, which together contribute to explain 62% of the variance contained in the dataset. There is no distinct separation between samples collected from ejaculates with resistant (blue dots) and sensitive (orange dots) sperm to 10% (*v*/*v*) autologous seminal plasma in extended semen at 144 h of storage.

**Figure 8:**
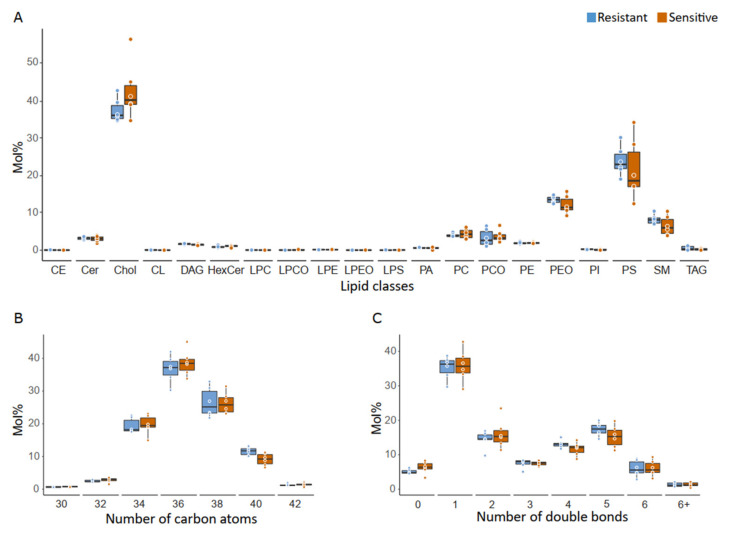
Lipid classes detected in seminal plasma, and profiles of total acyl chain length and unsaturation of nonstorage lipids. The mol% amounts of the different lipid features are presented as boxplots with dots. Each dot indicates a sample (*n* = 6 in each group). Values did not differ between sensitive and resistant samples (*p* > 0.05). (**A**) Lipid class composition. Mol% abundance of lipid classes in seminal plasma samples collected from ejaculates with resistant (blue bars) and sensitive (orange bars) sperm to 10% (*v*/*v*) autologous seminal plasma in extended semen at 144 h of storage. Prevalent lipid classes are cholesterol (Chol) and phosphatydilserine (PS). Classes appear in alphabetical order. The mole percent was calculated for all lipids. (**B**) Total acyl chain length profile. Lipids were regrouped according to the number of carbon atoms present in their acyl chains. The mole percent was calculated for sphingolipids, glycerophospholipids and diacylglycerides. Only length groups with a mean >1 mol l% are shown. Shorter chain lipids (lipids with 32 carbon atoms) are lowly abundant, as well as very long lipids (42 carbon atoms). The most represented group consists of lipids with 36 carbon atoms. (**C**) Unsaturation profile. Lipids were regrouped according to the number of double bonds present in their acyl chains. All species with more than six double bonds are indicated with “6+”. The mole percent was calculated for sphingolipids, glycerophospholipids and diacylglycerides. 0 = saturated lipids; 1 = mono-unsaturated lipids; 2 = di-unsaturated lipids; 3–6+ = poly-unsaturated lipids. Boar seminal plasma contains many mono-unsaturated phospho- and sphingolipids.

The lipidomes from ejaculates with resistant and sensitive sperm as detected at 144 h with 10% (*v*/*v*) SP appear to overlap when mapped by PCA (Figure 7), indicating that the amounts of lipid species characterising the two groups are similar. The lack of any clear-cut difference was also reflected by the lipid class composition (Figure 8A and Supplementary file “lipid_classes_SUPPLEMENTARY.xlsx”) and when testing lipid species (the dataset is provided in Supplementary file “dataset_filtered_SUPPLEMENTARY.xlsx”). Although the two groups are not differentiated by their lipidome, the high resolution of shotgun MS/MS allowed to give a more comprehensive profile of the lipids contained in the SP. The class composition showed a peculiar abundance in cholesterol (Chol) (sample mean 40 ± 6 mol%) and phosphatydilserine (PS) (sample mean 22.8 ± 6.2 mol%) (Figure 8A). The total acyl chain length (Figure 8B) and total acyl chain double bond profile (Figure 8C) calculated on glycerophospholipids, sphingolipids and diglycerols, indicates that SP is particularly enriched in monounsaturated species with 36 carbon atoms. The lipid classes that mainly contribute to this configuration are sphingolipids (Ceramides, Hexosyl-Ceramides, Sphingomyelins) together with phosphatidylserine.

## 3. Discussion

The present study shows that the differences in long-term semen preservability among individual boars can reside in the sperm’s tolerance to autologous SP and this could be associated with the differences in the abundance of three SP proteins. Moreover, the first shotgun analysis of the porcine SP lipidome does not indicate any association with semen preservability but is considered to initiate further research on the importance of the SP metabolome for sperm quality and fertility.

As shown previously for extended semen portions [3,4], the sensitivity of stored boar sperm to SP is time- and concentration-dependent affecting sperm motility, mitochondrial activity, and, to a lesser extent, the membrane integrity. Here, we tested effects of SP in standard semen doses as used in many European AI stations (18 × 10^6^ sperm/mL), taking into account that lower sperm concentrations render sperm more susceptible to the long-term effect of SP [3]. It is well-known that there are individual variations among boars concerning the preservability of sperm quality during in vitro storage [6]. Our results show that this can be partly attributed to a boar-specific sensitivity to higher concentrations of autologous SP during storage for 144 h. The findings of Caballero et al. [30] are in agreement with our study, showing a boar-specific detrimental effect of SP on highly diluted (0.3 × 10^6^ sperm/mL) semen in phosphate-buffered saline (PBS) during incubation for 2 h at 30 °C. The authors also showed that responses of spermatozoa to homologous SP (from another boar) varies between sires, indicating that the interaction between sperm and SP is complex. Nonetheless, the concept of specific components in SP being responsible for the beneficial or detrimental effects on spermatozoa and fertility is an ongoing subject of research in pigs as well as in other species [30,31]. In the present study, it is to note that in the majority of boars, SP-effects varied among ejaculates of the same boar. Boars were housed under the same conditions, and the ejaculates were collected in the same season and handled in the same standardised way so that influencing factors known to affect the quality of extended semen [32] or the SP composition [10] can be excluded as a source of variation.

Consistency of SP composition would be an important prerequisite for using biomarkers for predicting the semen preservation capacity and fertility. In contrast to blood, SP is an unregulated body fluid which is instantly released from several accessory genital glands during the ejaculation phase. Absence of the intracorporal balancing mechanism and progressive post-ejaculatory changes would explain a natural variation in SP components. Potential biomarkers, therefore, must have some robustness in their presence or expression levels.

Using LC-MS/MS and the *Sus scrofa* protein database, we were able to quantify 230 proteins, which is a lower number compared to the 390 proteins previously detected by Pérez-Patiño et al. [16]. The generally low number of identified proteins in the analysed boar SP samples can be explained by masking effects of the highly abundant and glycosylated spermadhesins. In order to prevent experimental variations and to keep the sample preparation as reproducible as possible, we decided not to deplete spermadhesins or to perform prefractionation prior to LC-MS analysis. Nonetheless, we found three candidate proteins which were less abundant in SP from SP-resistant compared to -sensitive semen samples. Among those, AWN is a candidate with biological evidence since it belongs to the heparin-binding spermadhesin family known to affect sperm quality in highly diluted semen [15]. AWN is highly abundant in the post sperm-rich fraction consisting mainly of secretion of the vesicular glands which make the bulk fraction of the ejaculate [18]. High voluminous ejaculates with low sperm concentrations result in AI doses with a relative high amount of SP, sometimes exceeding 10% (*v*/*v*). This would explain our observation that a lower sperm motility in stored semen doses is associated with higher amounts of SP and consequently higher amounts of AWN. This finding corresponds to a previous study reporting that the lowest-fertility boars had the highest amount of AWN-1 in their AI dose [24]. The granulin precursors are cysteine-rich glycoproteins with an MW of around 65 kDa, which are well conserved among pigs, humans, mice and rats [33], being expressed in somatic as well as male germ cells [34,35]. Progranulin was detected in human SP at a higher concentration (1700 ng/mL) compared to serum levels. The function of the granulin precursor in SP is yet unknown but an association with semen quality has been suggested in humans [36]. The 32 kDa cysteine protease, legumain, also referred to as asparaginyl endopeptidase, was the third differentially abundant candidate protein identified in the present study. This is in line with results of proteomic profiling in SP in dairy bulls, showing that overexpression of legumain as one of 29 proteins is associated with low fertility [37]. The function of legumain exceeds its protease activity [38]. However, a specific action of this protein on sperm function is unknown. It is to note that apart from AWN, there is no overlap with proteins associated with boar fertility or semen preservability identified in previous high-throughput proteomics [17,31]. As stated above, varying results could be explained by different workflows of protein separation and identification procedures [17]. Therefore, confirmation of proteomic results and further experiments (e.g., the depletion of these proteins) are needed to elucidate the functional role for maintenance of sperm viability during on vitro storage and ultimately fertility.

The lipidomes of sensitive and resistant boar sperm to 10% SP at 144 h displayed a similar composition. Interestingly, the class profile, comprising 20 lipid classes, which to the best of our knowledge is the most comprehensive published so far, showed cholesterol and phosphatydilserine as prevalent classes. The high abundance of cholesterol is in agreement with previous studies, where cholesterol was the main SP lipid being present in a concentration around 7 mg/dL [39] and accounting for one third of the total lipid content [40]; its content was also found to be higher in boars with hybrid genetics compared to other breeds [41]. It was shown that the SP-content of total lipids, cholesterol, phospholipids, polyunsaturated fatty acids (PUFA) and docosahexaenoic acid (DHA) is positively correlated to sperm motility, morphology and viability in raw semen [40]. In the context of semen preservation, the role of SP lipids, particularly cholesterol, in the antioxidative defence system [41], or protection of the plasma membrane from membrane disorder and loss of cholesterol during semen processing and chilling [42] might be relevant. The high amount of PS is instead newly reported in our study. While sphingomyelins (SM) were reported as a major component of porcine seminal fluid [43], in our analysis they covered 7 ± 2% of the lipidome when averaging all samples. The different methodologies used in previous studies do not allow for a direct comparison of the results. With the present study, we provide for the first time a comprehensive view of the lipidome of boar seminal plasma, with a resolution to the subspecies level for phospholipids and diacylglycerols.

In conclusion, we have shown that a boar specificity exists in the sensitivity of spermatozoa to SP. Three candidate proteins, granulin precursor, legumain and AWN precursor, were identified which might be indicative of the SP-tolerance of sperm during long-term storage. Moreover, a first lipidomic profile of boar SP is described, which identified cholesterol and PS as major lipids. The mechanism of how the three proteins influence sperm sensitivity to SP and the function of the major lipids in boar SP must be determined in further experiments.

## 4. Materials and Methods

### 4.1. Spermatological Analysis

#### 4.1.1. Chemicals and Media

All chemicals were of analytical grade and purchased from Sigma-Aldrich (Steinheim, Germany), Thermo Fisher Scientific (Waltham, MA, USA), Vector Laboratories (Burlingame, Kalifornien, USA), Enzo Life Science (Lörrach, Germany), Roth (Karlsruhe, Germany), SEVERA Electrophoresis (Heidelberg, Germany) and Beckman Coulter (Krefeld, Germany). Semen extender was obtained from Minitüb (Tiefenbach, Germany).

#### 4.1.2. Animals, Semen Processing and Seminal Plasma

Nineteen sexually mature and fertile boars (one to 5.5. years of age) of different breeds (Pietrain, Landrace, Duroc/Pietrain and Large White) housed in an AI station (BHZP, Rätzlingen, Germany) were used. At weekly intervals, between four and five ejaculates (entire semen without the bulbourethral gland secretion) per boar were collected by trained technicians from the AI station using the gloved hand method. All ejaculates fulfilled the standards for semen use in artificial insemination. One aliquot was diluted with a modified, pH-stabilised BTS extender (mBTS consisting of sodium bicarbonate 15.47 mM, HEPES 18.89 mM, tri-sodium citrate dihydrate 20.4 mM, potassium chloride 10.73 mM, glucose 168.71 mM, titriplex EDTA 2.74 mM, gentamicin 0.52 mM) to 18 × 10^6^ sperm/mL at a final volume of 100 mL. The remaining ejaculate was centrifuged in a prewarmed centrifuge at 2500× *g* for 3 min to harvest the sperm pellet. Every material to which semen constituents were exposed to was prewarmed to ensure isothermal conditions. To obtain the autologous SP, single aliquots of the supernatant of the first centrifugation were centrifuged twice at 3360× *g* for 10 min. The resulting supernatants were microscopically examined to verify that they were sperm-free. Immediately thereafter, aliquots of sperm-free SP were frozen on dry ice and then stored at −80 °C until proteome and lipidome analysis. The sperm concentration in the sperm pellet was determined with a Thoma-Hemocytometer. Sperm pellets were then diluted to 18 × 10^6^ sperm/mL to 100 mL in mBTS with either 10%, 5% or 1% (*v*/*v* in final volume) autologous SP. Semen samples were kept at room temperature for 90 min and then stored for 144 h in the dark at + 17 °C.

#### 4.1.3. Assessment of Sperm Motility

The computer-assisted semen analysis (CASA) was used to measure sperm motility. The CASA system AndroVision^®^ (Version 1.2, Minitüb, Tiefenbach, Germany) was equipped with an automated microscope warming stage, a digital camera (acA2440 – 75uc, Basler, Ahrensburg, Germany) and a TV adapter (60-C 1” 1.0×, Zeiss, Jena, Germany). An aliquot of stored semen was incubated at 38 °C in a water bath for 30 min under air before measurement. Samples were placed in a 20 µL counting chamber (Leja, Products, Nieuw Vennep, Netherlands) and at least 400 sperm/sample were recorded in four to five fields of view at 100 × (ocular 10×, objective 10×, camera adapter 1×) at a rate of 30 pictures per 0.5 s. The percentage of total motile spermatozoa was recorded. A spermatozoon was considered motile when its curved-line velocity was higher than 24 µm/s and its amplitude of lateral head displacement higher than 1 µm. After 72 h of semen storage, a thermic incubation was performed to assess the thermos-resistance of spermatozoa, which is a sensitive indicator of the quality of extended semen samples semen [44]. For this, an aliquot of 3 mL extended semen was incubated for 300 min at 38 °C in a water bath under air.

#### 4.1.4. Assessment of Sperm Membrane Integrity

The ‘Cyto Flex’ flow cytometer (Beckman Coulter, Krefeld, Germany) equipped with ‘CytExpert 2.3′ Software (Beckman Coulter, Krefeld, Germany) was used to assess the integrity of sperm plasma membranes and acrosomes. The flow cytometer was equipped with three lasers: laser 1 (488 nm, 50 mW), laser 2 (638 nm, 50 mW), laser 3 (405 nm, 80 mW). An amount of 50 µL diluted semen was added to 935 µL prewarmed HBS containing 5 µL propidium iodide (PI, stock solution of 200 μg/mL), 5 µL fluorescein conjugated peanut agglutinin (FITC-PNA, stock solution of 120 μg/mL) and 5 µL Hoechst (H) 33,342 (stock solution of 90 μg/mL). After 5 min incubation at 38 °C under air, the integrity of sperm plasma membranes and acrosomes was assessed in 10,000 individual spermatozoa with a medium flow rate of 30–40 µL/min. The H 33,342 stain was used to distinguish DNA-containing cells from dirt particles and was detected on fluorescence detector FL-1 (450/45 nm BP). The sperm population was gated by referring to the expected forward- and side-scatter. FITC-PNA was detected on fluorescence detector FL-2 (525/40 nm BP) and PI on FL-3 (610/20 nm BP).

#### 4.1.5. Assessment of Sperm Mitochondria Membrane Potential

The proportion of viable (plasma membrane intact, PI-negative) spermatozoa with hMMP was determined with the same flow cytometer as described above. For this, 1 mL diluted semen was incubated with 12 µL PI (stock solution of 1 mg/mL), 6 µL H 33,342 (stock solution of 150 μg/mL) and 1 µL JC-1 iodide (stock solution of 0.613 mM) for 15 min at 38 °C. Then, 200 µL incubated sperm suspension was added to 800 µL prewarmed HBS and measured with a medium flow rate of 30–40 µL/min. Individual sperm (*n* = 10,000) were detected on FL-1 (H 33342), FL-2 (JC-1), FL-4 (JC-1, 585/42 nm BP) and FL-5 (PI, 690/50 nm BP).

#### 4.1.6. Statistical Analysis

Sperm data were analysed using the statistic analysis software (SAS Enterprise Guide 7.1; SAS Institute, Cary, NC, USA). A two-factorial ANOVA with repeated measurements (PROC GLIMMIX) was used to identify differences between SP content at different storage periods. Data of the thermic incubation test were tested with the post hoc Bonferroni test (PROC MIXED). Pearson’s correlations were used to show relationships between parameters. A variance component estimation procedure was used to investigate the inter- and intra-boar variability. The intra-boar reliability was assessed by intraclass correlation (ICC). Measurements were classified as significant when *p* < 0.05. All data are expressed as mean ± standard deviation (SD).

### 4.2. Proteome Analysis

Proteome analysis was carried out at the Gene Centre of the University Munich, Germany. Seminal plasma samples from single ejaculates of twelve different boars were used. Ejaculates were grouped to “resistant” or “sensitive” samples based on the sperm quality of extended semen in 10% (*v*/*v*) SP at 144 h of storage. Six ejaculates, one each from Boars 2, 3, 5, 10, 17 and 18, showed high sperm quality (motility ≥ 65%, viability ≥ 65%, hMMP ≥ 90%) and were designated as “resistant”. Another six ejaculates, one each from Boars 4, 12, 14, 15, 16, 19, displayed low sperm quality in 10% SP, but a higher sperm quality in 1% SP (at least + 10 percentage points in motility, and at least + 5 percentage points in viability and hMMP); they were assigned to the “sensitive” group. Protein concentrations were determined using the Bradford [45] protein quantification method. Twenty µg of total protein was diluted in 10 µL NH_4_HCO_3_ and reduced using 1,4-Dithiothreitol (5 mM final concentration) at 37 °C for 30 min. To inhibit refolding of the proteins, cysteine residues were carbamidomethylated with iodacetamide at a final concentration of 15 mM for 30 min in the dark. Proteins were digested for 4 h at 37 °C using Lys-C (FUJIFILM Wako Chemicals Europe, Neuss, Germany) at an enzyme/substrate ratio of 1/100. A further digestion step using modified porcine trypsin (Promega, Madison, WI, USA) was performed at an enzyme/substrate ratio of 1/50 at 37 °C overnight. For mass spectrometry-based proteome analysis, an Ultimate 3000 nano-LC system (Thermo Fisher Scientific, Waltham, MA, USA) coupled to a Q Exactive HF-X mass spectrometer (Thermo Fisher Scientific) was used. One µg of each peptide sample was transferred at a flow rate of 20 µL/min to a trap column (Acclaim^®^ PepMap 100, 100 μm × 2cm, nanoViper C18, 5 μm, 100 Å, Thermo Scientific) and separated using a 50 cm EasySpray column (PepMap RSLC C18, 75 µm ID, 2 µm, Thermo Fisher Scientific) at a flow rate of 250 nL/min. As solvent A, 0.1% formic acid and as solvent B, 0.1% formic acid in acetonitrile were used. The chromatographic separation method consisted of consecutive gradients from 3% to 25% solvent B in 160 min, from 2 5% to 40% solvent B in 10 min and from 40% to 85% solvent B in 5 min, respectively. The mass spectrometer was operated in the data-dependent mode using cycles of one MS precursor scan (350 to 1600 m/z) at a resolution of 60 k, followed by up to 12 data-dependent MS/MS scans at a resolution of 15 k. For protein identification (FDR < 0.01) and for label-free quantification, MaxQuant (v. 1.6.1.0) [46] and the porcine subset of the RefSeq protein database were used. Principal component analysis, the Volcano plot and Students t-tests were performed using the Perseus software (V1.5.3.2) which is part of the MaxQuant software package.

### 4.3. Lipidome Analysis

#### 4.3.1. Lipid Extraction for Mass Spectrometry Lipidomics

Mass spectrometry-based lipid analysis was performed at Lipotype (Dresden, Germany) as previously described [47]. Seminal plasma samples of the same ejaculates as used for proteomic profiling were analysed. Lipids were extracted using a two-step chloroform/methanol procedure [48]. Samples were spiked with internal lipid standard mixture containing cardiolipin 16:1/15:0/15:0/15:0 (CL), ceramide 18:1;2/17:0 (Cer), diacylglycerol 17:0/17:0 (DAG), hexosylceramide 18:1;2/12:0 (HexCer), lyso-phosphatidate 17:0 (LPA), lyso-phosphatidylcholine 12:0 (LPC), lyso-phosphatidylethanolamine 17:1 (LPE), lyso-phosphatidylglycerol 17:1 (LPG), lyso-phosphatidylinositol 17:1 (LPI), lyso-phosphatidylserine 17:1 (LPS), phosphatidate 17:0/17:0 (PA), phosphatidylcholine 17:0/17:0 (PC), phosphatidylethanolamine 17:0/17:0 (PE), phosphatidylglycerol 17:0/17:0 (PG), phosphatidylinositol 16:0/16:0 (PI), phosphatidylserine 17:0/17:0, cholesterol ester 20:0 (CE), sphingomyelin 18:1;2/12:0;0, triacylglycerol 17:0/17:0/17:0 (TAG) and cholesterol D6. After extraction, the organic phase was transferred to an infusion plate and dried in a speed vacuum concentrator. The first step dry extract was resuspended in 7.5 mM ammonium acetate in chloroform/methanol/propanol (1:2:4, *v*:*v*:*v*) and the second step dry extract in 33% ethanol solution of methylamine in chloroform/methanol (0.003:5:1; *v*:*v*:*v*). All liquid handling steps were performed using Hamilton Robotics STARlet robotic platform with the anti droplet control feature for organic solvents pipetting.

#### 4.3.2. MS Data Acquisition

Samples were analysed by direct infusion on a QExactive mass spectrometer (Thermo Scientific) equipped with a TriVersa NanoMate ion source (Advion Biosciences, Harlow, UK). Samples were analysed in both positive and negative ion modes with a resolution of R_m/z = 200_ = 280,000 for MS and R_m/z = 200_ = 17,500 for MS/MS experiments, in a single acquisition. MS/MS was triggered by an inclusion list encompassing corresponding MS mass ranges scanned in 1 Da increments [49]. Both MS and MS/MS data were combined to monitor CE, DAG and TAG ions as ammonium adducts; PC, PC O-, as acetate adducts; and CL, PA, PE, PE O-, PG, PI and PS as deprotonated anions. MS only was used to monitor LPA, LPE, LPE O-, LPI and LPS as deprotonated anions; Cer, HexCer, SM, LPC and LPC O- as acetate adducts and cholesterol as ammonium adduct of an acetylated derivative [50].

#### 4.3.3. Data Analysis and Post-Processing

Data were analysed with in-house developed lipid identification software based on LipidXplorer [51,52]. Data post-processing and normalisation were performed using an in-house developed data management system. Only lipid identifications with a signal-to-noise ratio > 5 and a signal intensity five-fold higher than in corresponding blank samples were considered for further data analysis.

#### 4.3.4. Lipidomics Data Downstream Analysis

The lipidomics data matrix generated by Lipotype consisted of 568 lipids given in picomol per microgram of total protein. The data were filtered by removing all lipids that were measured in ≤ 15% of the samples (i.e., ≤2 samples). The filtered data matrix consisted of 427 lipids and was log2-transformed before undergoing PCA (scaled and centred). Variation between the two types of sperm-seminal fluid interaction (“resistant” versus “sensitive”) was explored by comparing the means of lipid classes and lipids regrouped according to the number of double bonds (unsaturation profile) and carbon atoms (total length profile) present in their acyl chains. Mean and standard deviation were calculated on the mol%-transformed data and statistically tested by means of either the Wilcoxon rank sum test or the Welch *t*-test, depending on the results of the Shapiro test for normality. All *p*-values were adjusted according to the Benjamini-Hochberg correction. Analyses were performed in R [53].

## Supplementary Materials

Supplementary materials can be found at https://www.mdpi.com/1422-0067/21/18/6474/s1.
