# Supplementary material for: Tolerance of Stored Boar Spermatozoa to Autologous Seminal Plasma: A Proteomic and Lipidomic Approach
Source: Int J Mol Sci. 2020 Sep 4;21(18):6474. doi: 10.3390/ijms21186474 (PMC7555833; doi:10.3390/ijms21186474)
Supplement: Supplementary file 1 [file ijms-21-06474-s001.zip › ijms-892100 suppl to convertion.pdf]

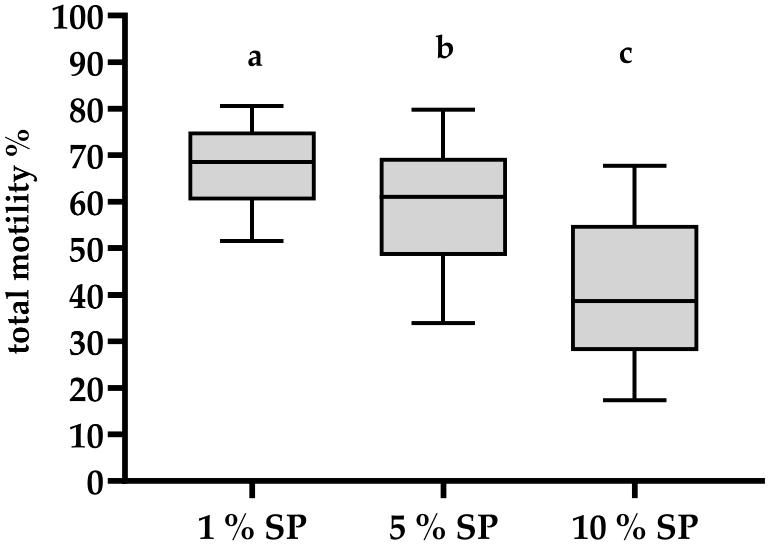

Figure S1. Motility after thermic incubation in semen doses with different concentrations of seminal plasma (SP). Box-whisker plots showing total motility (%) after subsequent thermic incubation at 38 °C for 300 min. in semen samples (n = 19 boars) stored for 72 h. Semen samples contain three different final concentrations of autologous SP: 1, 5 and 10 % (v/v). Total motility (%) of samples with the highest SP concentration was lower ($p < 0.001$) compared to semen samples with 1 % (v/v) and 5 % (v/v) SP. Semen samples with 5 % (v/v) SP showed a significant lower motility ($p < 0.01$) compared to semen samples with 1 % (v/v) SP. Different letters indicate significant differences ($p < 0.01$).

A

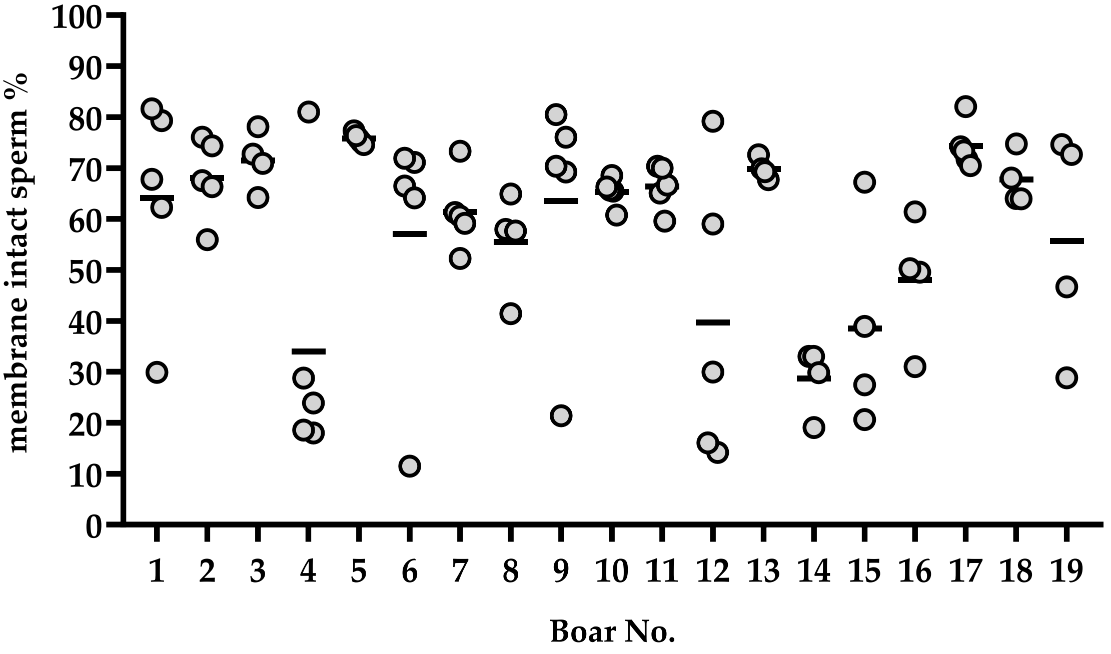

B

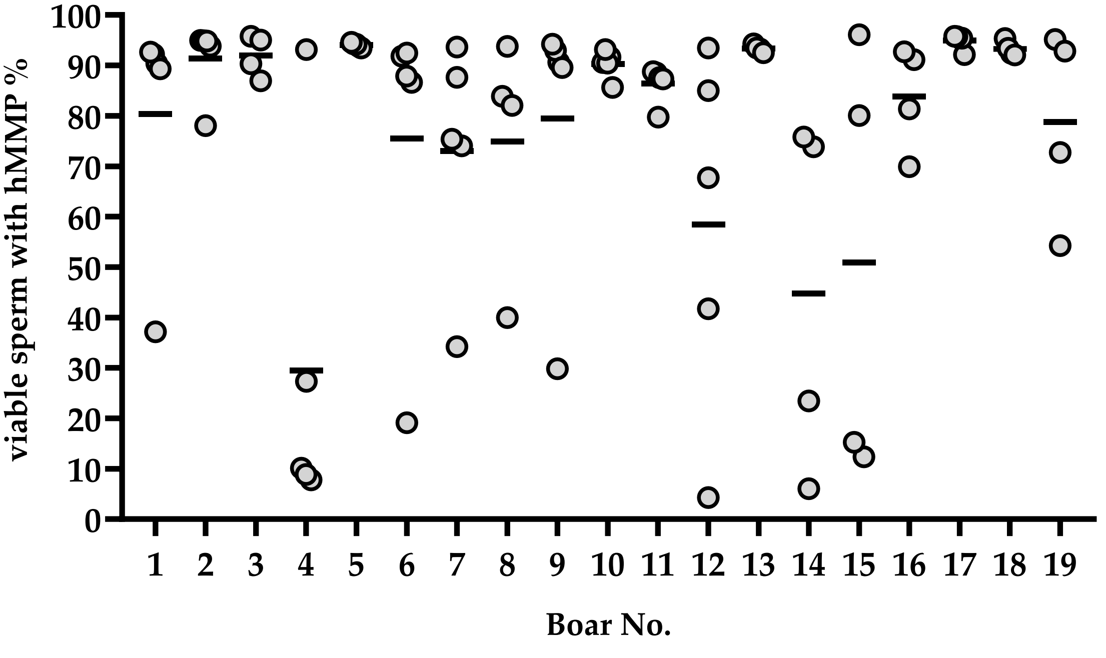

Figure S2. Sperm viability and mitochondria membrane potential after 144 h of storage in single semen doses containing 10 % (v/v) seminal plasma (SP). Scatterplots showing **(A)** membrane intact (propidium iodide negative and peanut agglutinin (PNA) negative) sperm (%), and **(B)** viable sperm (%) with high mitochondrial membrane potential (hMMP; propidium iodide negative sperm with J-aggregates) of semen samples with 10 % autologous SP collected from 19 boars (n = 4 – 5 ejaculates per boar) after 144 h of storage. Dots show the values measured in each sample and the bars show the mean for each boar.
